# 2-(Naphthalene-2-sulfonamido)-3-phenyl­propanoic acid

**DOI:** 10.1107/S1600536813000081

**Published:** 2013-01-09

**Authors:** Hafiz Mubashar-ur-Rehman, Muhammad Nadeem Arshad, Abdullah M. Asiri, Islam Ullah Khan, Muhammad Bilal

**Affiliations:** a Department of Chemistry, Materials Chemistry Laboratory, GC University, Lahore 54000, Pakistan; b Center of Excellence for Advanced Materials Research (CEAMR), Faculty of Science, King Abdulaziz University, PO Box 80203, Jeddah 21589, Saudi Arabia; c Chemistry Department, Faculty of Science, King Abdulaziz University, PO Box 80203, Jeddah 21589, Saudi Arabia; d Department of Chemistry, University of Engineering & Technology, Lahore 54000, Pakistan

## Abstract

In the title compound, C_19_H_17_NO_4_S, the phenyl ring and the naphthalene ring system are oriented at a dihedral angle of 4.12 (2)° and the mol­ecule adopts a U-shaped conformation. The C_c_—C—N—S (c = carb­oxy) torsion angle is 90.98 (15)°. In the crystal, mol­ecules are linked by O—H⋯O and N—H⋯O hydrogen bonds, resulting in (100) chains incorporating centrosymmetric *R* ^2^_2_(14) and *R* ^2^_2_(10) loops. Weak aromatic π–π stacking is also observed [centroid–centroid separations = 3.963 (2) and 3.932 (2) Å].

## Related literature

For the synthesis and related structures, see: Arshad *et al.* (2012 ▶); Khan *et al.* (2012 ▶).
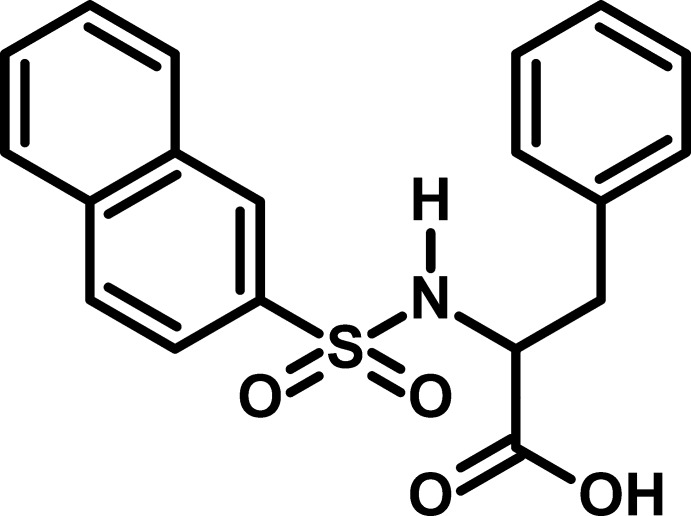


## Experimental

### 

#### Crystal data

C_19_H_17_NO_4_S
*M* *_r_* = 355.40
Monoclinic, 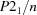

*a* = 8.0694 (2) Å
*b* = 15.2168 (4) Å
*c* = 14.0996 (3) Å
β = 92.505 (2)°
*V* = 1729.64 (7) Å^3^
*Z* = 4
Cu *K*α radiation
μ = 1.87 mm^−1^
*T* = 296 K
0.29 × 0.10 × 0.09 mm

#### Data collection

Agilent SuperNova (Dual, Cu at zero, Atlas) CCD diffractometer
Absorption correction: multi-scan (*CrysAlis PRO*; Agilent, 2012 ▶) *T*_min_ = 0.784, *T*_max_ = 1.000
13588 measured reflections
3486 independent reflections
2813 reflections with *I* > 2σ(*I*)
*R*_int_ = 0.026

#### Refinement

*R*[*F* ^2^ > 2σ(*F* ^2^)] = 0.041
*wR*(*F* ^2^) = 0.118
*S* = 1.03
3486 reflections
232 parameters
H atoms treated by a mixture of independent and constrained refinement
Δρ_max_ = 0.28 e Å^−3^
Δρ_min_ = −0.37 e Å^−3^

### 

Data collection: *CrysAlis PRO* (Agilent, 2012 ▶); cell refinement: *CrysAlis PRO*; data reduction: *CrysAlis PRO*; program(s) used to solve structure: *SHELXS97* (Sheldrick, 2008 ▶); program(s) used to refine structure: *SHELXL97* (Sheldrick, 2008 ▶); molecular graphics: *PLATON* (Spek, 2009 ▶); software used to prepare material for publication: *WinGX* (Farrugia, 2012 ▶) and *X-SEED* (Barbour, 2001 ▶).

## Supplementary Material

Crystal structure: contains datablock(s) I, global. DOI: 10.1107/S1600536813000081/hb7020sup1.cif

Structure factors: contains datablock(s) I. DOI: 10.1107/S1600536813000081/hb7020Isup2.hkl

Supplementary material file. DOI: 10.1107/S1600536813000081/hb7020Isup3.cml

Additional supplementary materials:  crystallographic information; 3D view; checkCIF report

## Figures and Tables

**Table 1:** Hydrogen-bond geometry (Å, °)

*D*—H⋯*A*	*D*—H	H⋯*A*	*D*⋯*A*	*D*—H⋯*A*
N1—H1*N*⋯O3^i^	0.80 (2)	2.18 (2)	2.964 (2)	164 (2)
O4—H1*O*⋯O2^ii^	0.88 (3)	1.90 (3)	2.772 (2)	174 (2)

Symmetry codes: (i) 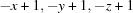; (ii) 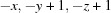.

**Fig. 1.**
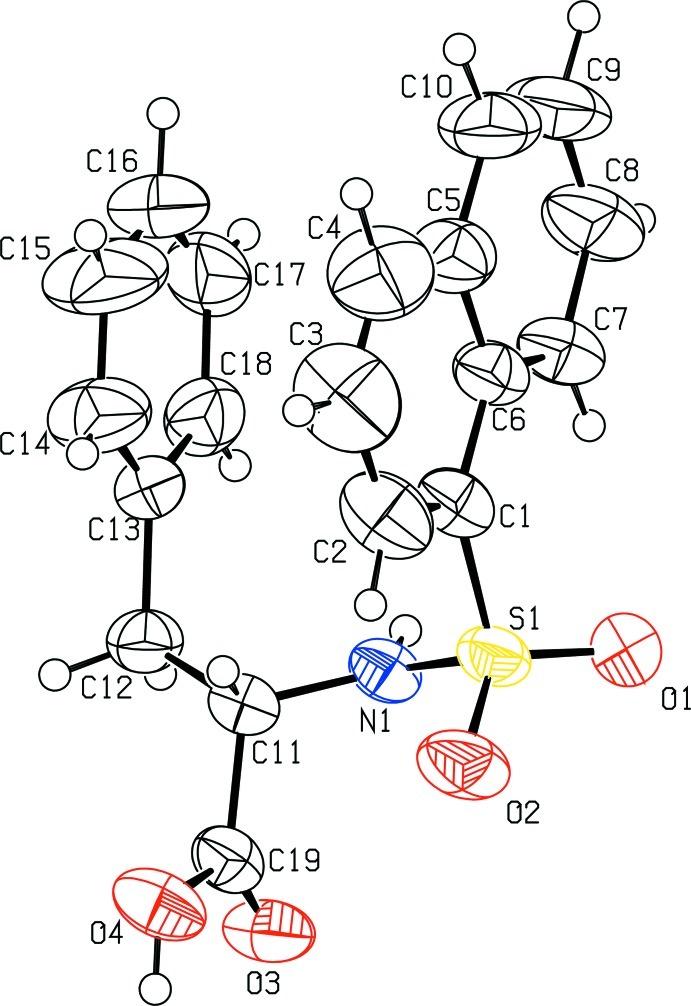
The molecular structure of C19H17NO4S with 50% probability of thermal ellipsoids.

**Fig. 2.**
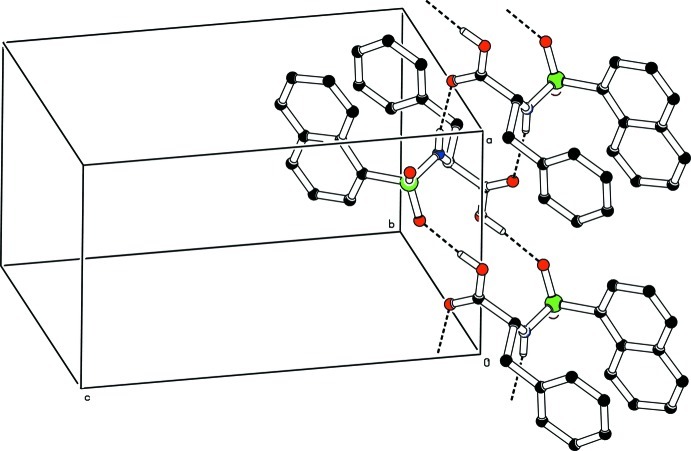
A perspective view showing O—H···O and N—H···O hydrogen bonds, drawn using dashed lines.

## supplementary crystallographic information

### Comment

In countinuation of our studies of the synthesis of sulfonamide derivatives of amino acids (Khan *et al.*, 2012) & (Arshad *et al.*, 2012) we now report the crystal structure of title compound.

In the structure of molecule the naphthalene and benzene rings are almost parallel, since the dihedral angle is as small as 4.12 (2)°. The sulfur (S1) atom as usual adopted the distorted tetrahedral geometry with O1-S1-O2 angle of 118.96 (9)°. The O—H···O type intermolecular hudrogen bonding connects the molecules to form inversion dimers and resulting in fourteen *R*^2^_2_(14) membered ring motifs. On the othe other hand, these dimers connected through another N—H···O type link to give ten *R*^2^_2_(10) membered ring motif and generates a long chain along the *a* axis direction (Table. 1, Fig. 2). The presence of π-π stacking interactions between aromatic rings is also observed: Cg1-Cg2 (Cg1: C1-C6; Cg2: C5-C10) with distances of 3.963 (2) Å (1 - x, 1 - y, 2 - z) and Cg2-Cg2 with distances of 3.932 (2) Å (1 - x, 1 -y , 2 - z).

### Experimental

The title compound was synthesised following the literature method (Arshad *et al.*, 2012) and recrystallized from methanol solution as colourless prisms by slow evaporation at room temperature.

### Refinement

All the C—H H-atoms were positioned with idealized geometry with C—H = 0.93 Å for aromatic, C—H = 0.97 Å for methylene & C—H = 0.98 Å for C_11_. H-atoms were refined as riding with *U*_iso_(H) = 1.2 *U*_eq_(C) for all H-atoms.

The N—H = 0.80 (2) & O—H = 0.88 (3) H-atoms were located with difference map *U*_iso_(H) = 1.2 *U*_eq_(N) and *U*_iso_(H) = 1.5 *U*_eq_(O).

### Crystal data

**Table:**

C_19_H_17_NO_4_S	*F*(000) = 744
*M**_r_* = 355.40	*D*_x_ = 1.365 Mg m^−^^3^
Monoclinic, *P*2_1_/*n*	Cu *K*α radiation, λ = 1.54184 Å
Hall symbol: -P 2yn	Cell parameters from 5975 reflections
*a* = 8.0694 (2) Å	θ = 3.1–74.6°
*b* = 15.2168 (4) Å	µ = 1.87 mm^−^^1^
*c* = 14.0996 (3) Å	*T* = 296 K
β = 92.505 (2)°	Prismatic, colorless
*V* = 1729.64 (7) Å^3^	0.29 × 0.10 × 0.09 mm
*Z* = 4	

### Data collection

**Table:**

Agilent SuperNova (Dual, Cu at zero, Atlas) CCD diffractometer	3486 independent reflections
Radiation source: SuperNova (Cu) X-ray Source	2813 reflections with *I* > 2σ(*I*)
Mirror monochromator	*R*_int_ = 0.026
ω scans	θ_max_ = 74.7°, θ_min_ = 4.3°
Absorption correction: multi-scan (*CrysAlis PRO*; Agilent, 2012)	*h* = −9→9
*T*_min_ = 0.784, *T*_max_ = 1.000	*k* = −18→18
13588 measured reflections	*l* = −17→17

### Refinement

**Table:**

Refinement on *F*^2^	Primary atom site location: structure-invariant direct methods
Least-squares matrix: full	Secondary atom site location: difference Fourier map
*R*[*F*^2^ > 2σ(*F*^2^)] = 0.041	Hydrogen site location: inferred from neighbouring sites
*wR*(*F*^2^) = 0.118	H atoms treated by a mixture of independent and constrained refinement
*S* = 1.03	*w* = 1/[σ^2^(*F*_o_^2^) + (0.0591*P*)^2^ + 0.4657*P*] where *P* = (*F*_o_^2^ + 2*F*_c_^2^)/3
3486 reflections	(Δ/σ)_max_ = 0.001
232 parameters	Δρ_max_ = 0.28 e Å^−^^3^
0 restraints	Δρ_min_ = −0.37 e Å^−^^3^

### Special details

**Table:**

Geometry. All esds (except the esd in the dihedral angle between two l.s. planes) are estimated using the full covariance matrix. The cell esds are taken into account individually in the estimation of esds in distances, angles and torsion angles; correlations between esds in cell parameters are only used when they are defined by crystal symmetry. An approximate (isotropic) treatment of cell esds is used for estimating esds involving l.s. planes.
Refinement. Refinement of F^2^ against ALL reflections. The weighted R-factor wR and goodness of fit S are based on F^2^, conventional R-factors R are based on F, with F set to zero for negative F^2^. The threshold expression of F^2^ > 2sigma(F^2^) is used only for calculating R-factors(gt) etc. and is not relevant to the choice of reflections for refinement. R-factors based on F^2^ are statistically about twice as large as those based on F, and R- factors based on ALL data will be even larger.

### Fractional atomic coordinates and isotropic or equivalent isotropic displacement parameters (Å^2^)

**Table:**

	*x*	*y*	*z*	*U*_iso_*/*U*_eq_	
S1	0.31495 (6)	0.52701 (3)	0.68873 (3)	0.05113 (16)	
O1	0.4041 (2)	0.60690 (9)	0.70303 (11)	0.0687 (4)	
O2	0.14144 (17)	0.53082 (11)	0.66184 (11)	0.0707 (4)	
O3	0.24930 (18)	0.47490 (10)	0.41829 (10)	0.0647 (4)	
O4	0.05674 (17)	0.39149 (11)	0.48022 (11)	0.0671 (4)	
N1	0.40035 (19)	0.47399 (10)	0.60505 (10)	0.0470 (4)	
C1	0.3324 (2)	0.46054 (13)	0.79139 (13)	0.0521 (4)	
C2	0.1937 (3)	0.41529 (18)	0.81402 (16)	0.0749 (6)	
H2	0.0955	0.4223	0.7778	0.090*	
C3	0.1997 (4)	0.3582 (2)	0.8920 (2)	0.1020 (10)	
H3	0.1058	0.3263	0.9066	0.122*	
C4	0.3406 (4)	0.3492 (2)	0.9463 (2)	0.0980 (9)	
H4	0.3416	0.3119	0.9985	0.118*	
C5	0.4860 (3)	0.39491 (16)	0.92563 (15)	0.0706 (6)	
C6	0.4857 (2)	0.45189 (13)	0.84526 (13)	0.0524 (4)	
C7	0.6347 (3)	0.49537 (16)	0.82581 (15)	0.0623 (5)	
H7	0.6387	0.5319	0.7731	0.075*	
C8	0.7732 (3)	0.4843 (2)	0.88355 (19)	0.0839 (8)	
H8	0.8705	0.5135	0.8697	0.101*	
C9	0.7708 (4)	0.4296 (2)	0.9636 (2)	0.0969 (9)	
H9	0.8656	0.4234	1.0030	0.116*	
C10	0.6320 (4)	0.3866 (2)	0.98330 (18)	0.0909 (9)	
H10	0.6320	0.3502	1.0363	0.109*	
C11	0.3160 (2)	0.39725 (12)	0.56401 (12)	0.0454 (4)	
H11	0.2472	0.3704	0.6117	0.054*	
C12	0.4398 (2)	0.32926 (13)	0.53018 (13)	0.0535 (4)	
H12A	0.3793	0.2812	0.4996	0.064*	
H12B	0.5084	0.3561	0.4834	0.064*	
C13	0.5497 (2)	0.29330 (12)	0.61016 (13)	0.0516 (4)	
C14	0.4856 (4)	0.23896 (18)	0.6765 (2)	0.0874 (8)	
H14	0.3741	0.2234	0.6713	0.105*	
C15	0.5838 (5)	0.2070 (2)	0.7509 (2)	0.1202 (12)	
H15	0.5374	0.1712	0.7961	0.144*	
C16	0.7457 (5)	0.2268 (2)	0.7590 (2)	0.1056 (11)	
H16	0.8113	0.2041	0.8091	0.127*	
C17	0.8139 (3)	0.28037 (19)	0.6937 (2)	0.0895 (9)	
H17	0.9262	0.2943	0.6992	0.107*	
C18	0.7150 (3)	0.31431 (15)	0.61838 (17)	0.0665 (5)	
H18	0.7613	0.3511	0.5739	0.080*	
C19	0.2050 (2)	0.42710 (13)	0.47926 (13)	0.0491 (4)	
H1N	0.499 (3)	0.4785 (14)	0.6034 (16)	0.059*	
H1O	−0.004 (3)	0.4135 (16)	0.4325 (18)	0.074*	

### Atomic displacement parameters (Å^2^)

**Table:**

	*U*^11^	*U*^22^	*U*^33^	*U*^12^	*U*^13^	*U*^23^
S1	0.0438 (3)	0.0605 (3)	0.0478 (3)	0.00944 (19)	−0.01179 (18)	−0.00627 (19)
O1	0.0815 (10)	0.0540 (8)	0.0685 (9)	0.0020 (7)	−0.0201 (8)	−0.0057 (7)
O2	0.0465 (8)	0.0973 (11)	0.0667 (9)	0.0235 (7)	−0.0148 (6)	−0.0138 (8)
O3	0.0578 (9)	0.0813 (10)	0.0536 (8)	−0.0026 (7)	−0.0119 (6)	0.0181 (7)
O4	0.0452 (8)	0.0972 (11)	0.0578 (8)	−0.0087 (7)	−0.0101 (6)	0.0008 (8)
N1	0.0368 (7)	0.0587 (9)	0.0449 (8)	−0.0018 (7)	−0.0044 (6)	−0.0024 (6)
C1	0.0458 (10)	0.0687 (12)	0.0417 (9)	−0.0002 (8)	−0.0003 (7)	−0.0060 (8)
C2	0.0590 (13)	0.1014 (18)	0.0645 (13)	−0.0162 (12)	0.0052 (10)	−0.0047 (12)
C3	0.097 (2)	0.123 (2)	0.0877 (19)	−0.0394 (19)	0.0219 (17)	0.0136 (18)
C4	0.120 (3)	0.106 (2)	0.0686 (16)	−0.0131 (19)	0.0126 (16)	0.0233 (15)
C5	0.0856 (16)	0.0774 (14)	0.0485 (11)	0.0091 (12)	−0.0012 (10)	0.0035 (10)
C6	0.0518 (11)	0.0639 (11)	0.0412 (9)	0.0067 (8)	−0.0035 (7)	−0.0039 (8)
C7	0.0483 (11)	0.0830 (14)	0.0547 (11)	0.0030 (10)	−0.0083 (9)	0.0009 (10)
C8	0.0529 (14)	0.117 (2)	0.0803 (16)	0.0082 (13)	−0.0203 (12)	−0.0049 (15)
C9	0.085 (2)	0.127 (2)	0.0751 (17)	0.0339 (19)	−0.0363 (15)	−0.0053 (16)
C10	0.112 (2)	0.102 (2)	0.0563 (13)	0.0313 (18)	−0.0201 (14)	0.0092 (13)
C11	0.0428 (9)	0.0535 (10)	0.0394 (8)	−0.0005 (7)	−0.0033 (7)	0.0017 (7)
C12	0.0599 (11)	0.0562 (10)	0.0439 (9)	0.0092 (9)	−0.0029 (8)	0.0030 (8)
C13	0.0578 (11)	0.0459 (9)	0.0503 (10)	0.0072 (8)	−0.0056 (8)	0.0027 (8)
C14	0.0871 (18)	0.0854 (17)	0.0880 (17)	−0.0149 (14)	−0.0151 (14)	0.0390 (14)
C15	0.137 (3)	0.119 (3)	0.102 (2)	−0.004 (2)	−0.026 (2)	0.065 (2)
C16	0.136 (3)	0.093 (2)	0.0835 (19)	0.028 (2)	−0.0475 (19)	0.0104 (16)
C17	0.0658 (16)	0.0867 (18)	0.113 (2)	0.0183 (13)	−0.0335 (15)	−0.0249 (17)
C18	0.0580 (12)	0.0615 (12)	0.0798 (14)	0.0071 (10)	−0.0015 (10)	−0.0006 (11)
C19	0.0409 (9)	0.0603 (11)	0.0456 (9)	0.0028 (8)	−0.0032 (7)	−0.0083 (8)

### Geometric parameters (Å, º)

**Table:**

S1—O1	1.4226 (15)	C8—C9	1.404 (4)
S1—O2	1.4356 (14)	C8—H8	0.9300
S1—N1	1.6090 (16)	C9—C10	1.337 (4)
S1—C1	1.766 (2)	C9—H9	0.9300
O3—C19	1.193 (2)	C10—H10	0.9300
O4—C19	1.314 (2)	C11—C12	1.529 (2)
O4—H1O	0.88 (3)	C11—C19	1.531 (2)
N1—C11	1.459 (2)	C11—H11	0.9800
N1—H1N	0.80 (2)	C12—C13	1.507 (2)
C1—C2	1.364 (3)	C12—H12A	0.9700
C1—C6	1.430 (3)	C12—H12B	0.9700
C2—C3	1.401 (4)	C13—C14	1.367 (3)
C2—H2	0.9300	C13—C18	1.372 (3)
C3—C4	1.350 (4)	C14—C15	1.376 (4)
C3—H3	0.9300	C14—H14	0.9300
C4—C5	1.405 (4)	C15—C16	1.340 (5)
C4—H4	0.9300	C15—H15	0.9300
C5—C10	1.408 (4)	C16—C17	1.364 (5)
C5—C6	1.427 (3)	C16—H16	0.9300
C6—C7	1.410 (3)	C17—C18	1.399 (3)
C7—C8	1.364 (3)	C17—H17	0.9300
C7—H7	0.9300	C18—H18	0.9300
			
O1—S1—O2	118.96 (9)	C9—C10—C5	121.6 (2)
O1—S1—N1	107.60 (9)	C9—C10—H10	119.2
O2—S1—N1	105.70 (8)	C5—C10—H10	119.2
O1—S1—C1	110.58 (9)	N1—C11—C12	111.45 (15)
O2—S1—C1	106.34 (10)	N1—C11—C19	108.61 (14)
N1—S1—C1	107.01 (9)	C12—C11—C19	108.98 (14)
C19—O4—H1O	108.0 (16)	N1—C11—H11	109.3
C11—N1—S1	119.01 (12)	C12—C11—H11	109.3
C11—N1—H1N	120.3 (16)	C19—C11—H11	109.3
S1—N1—H1N	115.9 (16)	C13—C12—C11	112.56 (15)
C2—C1—C6	122.0 (2)	C13—C12—H12A	109.1
C2—C1—S1	116.37 (16)	C11—C12—H12A	109.1
C6—C1—S1	121.61 (15)	C13—C12—H12B	109.1
C1—C2—C3	119.8 (2)	C11—C12—H12B	109.1
C1—C2—H2	120.1	H12A—C12—H12B	107.8
C3—C2—H2	120.1	C14—C13—C18	118.6 (2)
C4—C3—C2	120.5 (3)	C14—C13—C12	120.2 (2)
C4—C3—H3	119.8	C18—C13—C12	121.15 (19)
C2—C3—H3	119.8	C13—C14—C15	120.8 (3)
C3—C4—C5	121.5 (3)	C13—C14—H14	119.6
C3—C4—H4	119.2	C15—C14—H14	119.6
C5—C4—H4	119.2	C16—C15—C14	120.8 (3)
C4—C5—C10	121.4 (2)	C16—C15—H15	119.6
C4—C5—C6	119.5 (2)	C14—C15—H15	119.6
C10—C5—C6	119.1 (2)	C15—C16—C17	119.8 (3)
C7—C6—C5	117.90 (19)	C15—C16—H16	120.1
C7—C6—C1	125.40 (18)	C17—C16—H16	120.1
C5—C6—C1	116.69 (19)	C16—C17—C18	120.0 (3)
C8—C7—C6	120.6 (2)	C16—C17—H17	120.0
C8—C7—H7	119.7	C18—C17—H17	120.0
C6—C7—H7	119.7	C13—C18—C17	119.9 (2)
C7—C8—C9	120.9 (3)	C13—C18—H18	120.0
C7—C8—H8	119.5	C17—C18—H18	120.0
C9—C8—H8	119.5	O3—C19—O4	124.08 (17)
C10—C9—C8	119.9 (2)	O3—C19—C11	124.07 (16)
C10—C9—H9	120.0	O4—C19—C11	111.81 (17)
C8—C9—H9	120.0		
			
O1—S1—N1—C11	−168.97 (13)	C1—C6—C7—C8	178.1 (2)
O2—S1—N1—C11	−40.88 (15)	C6—C7—C8—C9	−0.1 (4)
C1—S1—N1—C11	72.17 (15)	C7—C8—C9—C10	1.0 (5)
O1—S1—C1—C2	141.61 (17)	C8—C9—C10—C5	−0.4 (5)
O2—S1—C1—C2	11.1 (2)	C4—C5—C10—C9	179.7 (3)
N1—S1—C1—C2	−101.49 (18)	C6—C5—C10—C9	−1.1 (4)
O1—S1—C1—C6	−41.01 (18)	S1—N1—C11—C12	−148.96 (13)
O2—S1—C1—C6	−171.48 (15)	S1—N1—C11—C19	90.98 (15)
N1—S1—C1—C6	75.89 (17)	N1—C11—C12—C13	62.6 (2)
C6—C1—C2—C3	−0.1 (4)	C19—C11—C12—C13	−177.57 (16)
S1—C1—C2—C3	177.2 (2)	C11—C12—C13—C14	70.3 (3)
C1—C2—C3—C4	1.5 (5)	C11—C12—C13—C18	−109.5 (2)
C2—C3—C4—C5	−1.3 (5)	C18—C13—C14—C15	1.0 (4)
C3—C4—C5—C10	178.9 (3)	C12—C13—C14—C15	−178.8 (3)
C3—C4—C5—C6	−0.3 (4)	C13—C14—C15—C16	−1.5 (6)
C4—C5—C6—C7	−178.9 (2)	C14—C15—C16—C17	1.0 (6)
C10—C5—C6—C7	1.9 (3)	C15—C16—C17—C18	−0.1 (5)
C4—C5—C6—C1	1.6 (3)	C14—C13—C18—C17	−0.1 (3)
C10—C5—C6—C1	−177.6 (2)	C12—C13—C18—C17	179.6 (2)
C2—C1—C6—C7	179.1 (2)	C16—C17—C18—C13	−0.3 (4)
S1—C1—C6—C7	1.9 (3)	N1—C11—C19—O3	47.5 (2)
C2—C1—C6—C5	−1.4 (3)	C12—C11—C19—O3	−74.1 (2)
S1—C1—C6—C5	−178.65 (15)	N1—C11—C19—O4	−134.51 (16)
C5—C6—C7—C8	−1.4 (3)	C12—C11—C19—O4	103.90 (18)

### Hydrogen-bond geometry (Å, º)

**Table:**

*D*—H···*A*	*D*—H	H···*A*	*D*···*A*	*D*—H···*A*
N1—H1*N*···O3^i^	0.80 (2)	2.18 (2)	2.964 (2)	164 (2)
O4—H1*O*···O2^ii^	0.88 (3)	1.90 (3)	2.772 (2)	174 (2)

Symmetry codes: (i) −*x*+1, −*y*+1, −*z*+1; (ii) −*x*, −*y*+1, −*z*+1.
